# Stability Study and Identification of Degradation Products of Caffeoylgluconic Acid Derivatives from Fructus Euodiae

**DOI:** 10.3390/molecules23081975

**Published:** 2018-08-08

**Authors:** Huijuan Yu, Jing Yang, Jiamin Ding, Ying He, Zhenzuo Jiang, Xin Chai, Yuefei Wang

**Affiliations:** Tianjin State Key Laboratory of Modern Chinese Medicine, Tianjin University of Traditional Chinese Medicine, Tianjin 300193, China; yuhuijuan_2017@126.com (H.Y.); 13848106684@163.com (J.D.); heying441423@163.com (Y.H.); zhenzuojiang@hotmail.com (Z.J.); wangyf0622@tjutcm.edu.cn (Y.W.)

**Keywords:** Fructus Euodiae, UPLC-DAD/ESI-Q-TOF MS, caffeoylgluconic acids, stability, degradation

## Abstract

Caffeoylgluconic acid derivatives are characteristic constituents isolated from the aqueous extract of Fructus Euodiae. In this research focusing on caffeoylgluconic acid derivatives, *trans*-caffeoyl-6-*O*-d-gluconic acid (CGA), *trans*-caffeoyl-6-*O*-d-gluconic acid methyl ester (CGA-ME), and *trans*-caffeoyl-6-*O*-d-glucono-*γ*-lactone (CGA-LT), a systematic study of stability was performed under different temperatures and pH levels by ultra performance liquid chromatography-diode array detector (UPLC-DAD) and ultra performance liquid chromatography-diode array detector/electrospray ionization-quadrupole-time of flight mass spectrometry (UPLC-DAD/ESI-Q-TOF MS). From the concentration–time curves and sensitivity index (SeI), it was found that compared to CGA, which is inert to the variation of temperature and pH in the tested range, CGA-ME and CGA-LT were more sensitive, with stabilities more likely to be influenced by temperature. Considering the stability index (StI), the integrated stability of CGA was the best, and that of CGA-ME was the worst. In terms of the quasi-molecular and fragment ions of the tested compounds, the degradation products were identified or tentatively characterized, which could shed light on the degradation pathways. CGA-ME and CGA-LT were easily converted to CGA by hydrolytic reaction, all of which were susceptible to the formation of isomers. This study elucidated the degradation mechanism of caffeoylgluconic acid derivatives, contributing to better guidance on manufacturing and controlling the quality of drugs.

## 1. Introduction

Traditional Chinese medicines (TCMs) are in widespread use around the world as complementary therapy, increasingly concerned in Western nations [1,2]. In recent years, the stability of active compounds from TCMs has attracted especially extensive attention, having a vital influence on their quality, safety, and efficacy (QSE) [3,4,5,6,7]. Drug regulatory agencies around the world have drafted guidelines for manufacturing and controlling the QSE of drugs. Drug stability and degradation studies are an integral part of drug development. Stability testing is an important approach to evaluate the quality of drug substances and products, which may vary with time under the influence of temperature, humidity, light, etc. [8]. The International Conference on Harmonization (ICH) has released a series of guidelines on stability studies of pharmaceuticals [9]. In order to comply with the stringent requirements of regulatory guidelines, quality control and stability studies have taken a front seat in the TCM development process. Unlike synthetic drugs, it is well known that TCMs and their preparations generally exert their therapeutic effects through the synergic effects of multiple active ingredients [5,10,11]. Therefore, it is much more complicated to guarantee QSE under the influence of various storage conditions for a TCM product. The major challenges, namely the chemical complexity and variability in the chemical composition of the raw material, have posed an impediment for the stability testing of TCM products [12]. Consequently, the systematic study of active compounds and their stability has become an indispensable and urgent task, which would contribute to improvements in manufacturing and controlling the quality of TCMs.

The nearly ripe fruits of *Euodia rutaecarpa* (Juss.) Benth., *Euodia rutaecarpa* (Juss.) Benth. var. *officinalis* (Dode) Huang, and *Euodia rutaecarpa* (Juss.) Benth. var. *bodinieri* (Dode) Huang, known as Wuzhuyu, have been used to treat headache, gastrointestinal disorders, abdominal pain during menstruation, vomiting, and diarrhea [13]. Previous studies focused on the fat-soluble substances of these fruits, yet the water-soluble substances were not paid enough attention. However, patients usually take a decoction of Fructus Euodiae (FE) for clinical use. In our previous study, a large quantity of phenolic acids, including caffeoylgluconic, feruloylgluconic, and chlorogenic acid derivatives, were identified from the aqueous extract of FE using the UPLC-Q-TOF MS technique. Three caffeoylgluconic acid derivatives, *trans*-caffeoyl-6-*O*-d-gluconic acid (CGA), *trans*-caffeoyl-6-*O*-d-gluconic acid methyl ester (CGA-ME), and *trans*-caffeoyl-6-*O*-d-glucono-*γ*-lactone (CGA-LT) (Figure S1), were isolated from the aqueous extract of FE with the guidance of UPLC-Q-TOF MS analytical results [14]. Caffeoylgluconic acid derivatives, which belong to phenolic acids, were rarely reported in the literature [14,15]. Modern research suggested that phenolic acids have many pharmacological effects, such as dilating blood vessels, antioxidant and anti-inflammatory activity, analgesia, and antibiosis [16,17,18]. During the process of isolating the three caffeoylgluconic acid derivatives, it was found that the compounds were unstable to temperature and pH extremes and could convert to each other; thus, a long and arduous journey was followed to obtain the pure compounds. Since there were numerous caffeoylgluconic acid derivatives in the aqueous extract of FE, it was necessary to investigate their stability to guide the separation and further pharmaceutical study of these compounds.

Different analytical techniques, such as thin layer chromatography (TLC), high performance liquid chromatography (HPLC), HPLC based on hydrophilic interaction chromatography-electrospray ionization-mass spectrometry (HILIC-ESI-MS), electrospray ionization Fourier transform ion cyclotron resonance tandem mass spectrometry (ESI-FTICR-MS), liquid chromatography coupled with mass spectrometry (LC-MS), and nuclear magnetic resonance (NMR), have been established to evaluate the stability of the characteristic ingredients of TCMs and track the possible degradation products to clarify the degradation pathways [19,20,21,22,23,24,25,26]. Among these techniques, UPLC-UV is the most comprehensive method with low cost, high sensitivity, and favorable separating power that can be employed to detect the targeted compounds using UV absorption. In addition, UPLC-MS provides a powerful supplementary tool for profiling the targeted compounds and degradation products, which can afford accurate information of quasi-molecular and fragment ions for the identification of the compounds of interest [27,28,29].

In the present study, the stability of caffeoylgluconic acid derivatives in aqueous solutions at different temperatures and pH values was studied by UPLC analysis. Furthermore, a UPLC-DAD/ESI-Q-TOF MS method was established to study the degradation products of the three caffeoylgluconic acids derivatives. The structures of the degradation products and formation mechanisms were deduced according to the mass information of the quasi-molecular and fragment ions of the degradation products.

## 2. Results and Discussion

### 2.1. Effect of Temperature and pH on Stability of CGA, CGA-ME, and CGA-LT

**Figure 1:**
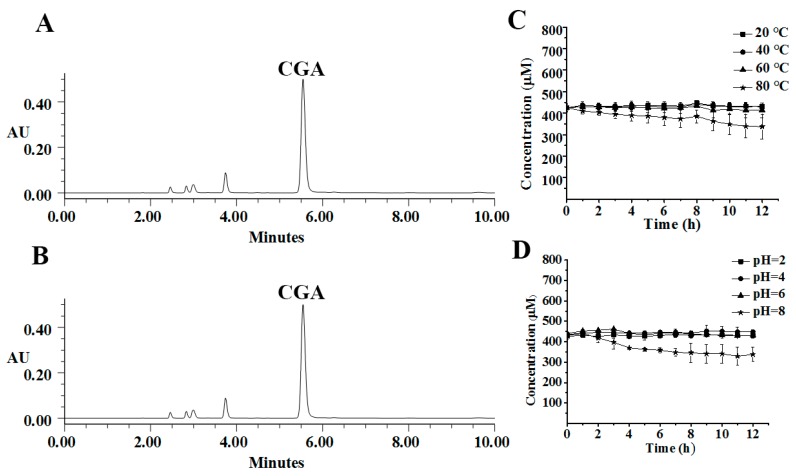
UPLC representative chromatograms of *trans*-caffeoyl-6-*O*-d-gluconic acid (CGA) in the exposed solution at 80 °C (**A**) and pH 8 (**B**) after 12 h; Time–concentration curves of CGA at different temperatures (**C**) and pH values (**D**).

**Figure 2:**
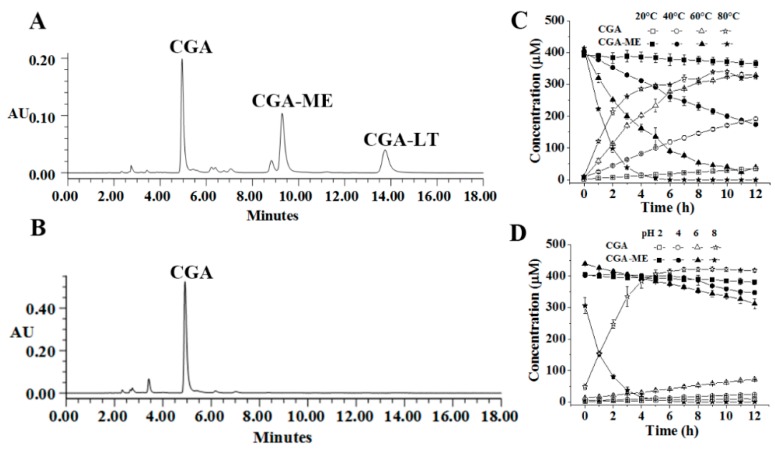
Representative UPLC chromatograms of *trans*-caffeoyl-6-*O*-d-gluconic acid methyl ester (CGA-ME) in the exposed solution at 40 °C (**A**) and pH 8 (**B**) after 12 h; Time–concentration curves of CGA-ME and CGA at different temperatures (**C**) and pH values (**D**).

**Figure 3:**
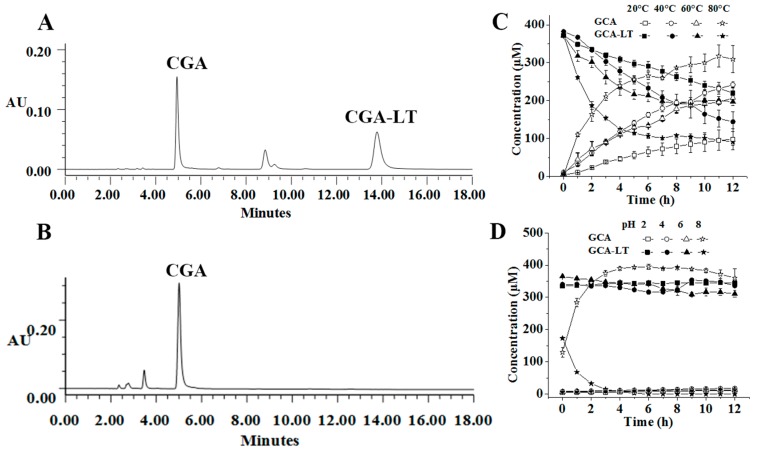
Representative UPLC chromatograms of *trans*-caffeoyl-6-*O*-d-glucono-*γ*-lactone (CGA-LT) in the exposed solution at 40 °C (**A**) and pH 8 (**B**) after 12 h; Time–concentration curves of CGA-LT and CGA at different temperatures (**C**) and pH values (**D**).

CGA, CGA-ME (methyl-esterified derivative of CGA), and CGA-LT (lactonized derivative of CGA), were the representative compounds existing in FE. A stability study of these compounds was systematically undertaken. Shown in Figure 1, Figure 2 and Figure 3 are the representative chromatograms and variation curves of concentration with time.

As for the stability of CGA, CGA-ME, and CGA-LT exposed to different temperatures (20, 40, 60, and 80 °C), with increasing temperature, CGA-ME and CGA-LT were degraded and converted into CGA at an accelerating speed; this was not applicable to CGA, which was relatively stable and inert to temperature variations. Especially surprising was that CGA-ME and CGA-LT rapidly converted into CGA in alkaline medium rather than in acidic medium, which is displayed in Figure 2D and Figure 3D. Different to CGA-ME and CGA-LT, CGA was slightly degraded in alkaline medium. Regardless of the temperature or pH, with time, the conversion from CGA-ME and CGA-LT to CGA was aggravated, which was mediated by a hydrolysis reaction [30,31,32].

### 2.2. Evaluation of Stability Index (StI) and Sensitivity Index (SeI) of CGA, CGA-ME, and CGA-LT

In order to comprehensively evaluate the degradation susceptibility and integrated stability of CGA, CGA-ME, and CGA-LT under different temperatures and pH values, the sensitivity index (SeI) and stability index (StI) were established and applied in this study. 

**Figure 4:**
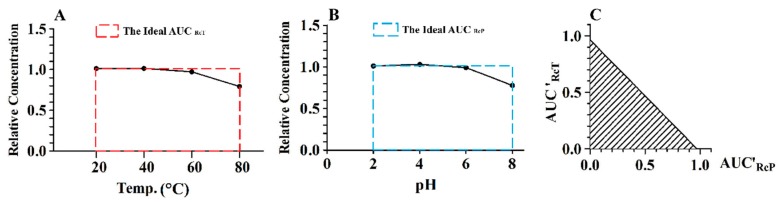
Relative concentration–temperature curve of CGA (**A**); relative concentration–pH curve of CGA (**B**); calculation model of sensitivity index (SeI) and stability index (StI) for CGA (**C**).

CGA is taken as an example to introduce the data processing as displayed in Figure 4; the relative concentrations at the different temperatures and pH values were obtained by dividing the CGA concentrations at 12 h of exposure to the different temperatures and pH values by the prepared concentration of CGA. The area under the relative concentration–temperature curve (AUC_RcT_) and the area under the relative concentration–pH curve (AUC_RcP_) were calculated; these were respectively divided by the ideal AUC_RcT_ and AUC_RcP_, and the results were nominated as AUC’_RcT_ and AUC’_RcP_, respectively. The computational formulae for SeI and StI are listed as follows.

(1) $$\mathrm{SeI}=\frac{{\mathrm{AUC}\prime }_{\mathrm{RcT}}}{{\mathrm{AUC}\prime }_{\mathrm{RcP}}}\;$$

(2) $$\mathrm{StI}={\mathrm{AUC}\prime }_{\mathrm{RcT}}\times {\mathrm{AUC}\prime }_{\mathrm{RcP}}/2\;$$

**Figure 5:**
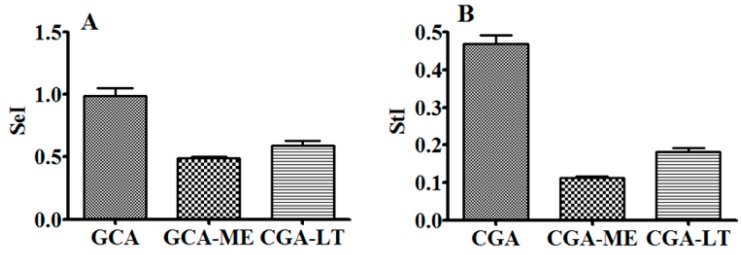
SeI (**A**) and StI (**B**) of CGA, CGA-ME, and CGA-LT (*n* = 3).

Displayed in Figure 5 are the SeI and StI results of CGA, CGA-ME, and CGA-LT. The SeI values of CGA, CGA-ME, and CGA-LT were 0.9926 (±0.059), 0.4883 (±0.018), and 0.5900 (±0.042), respectively. When the SeI of a compound was above 1, it was speculated that the tested compound was more sensitive to the variation of pH than that of temperature, and vice versa. The StI values of CGA, CGA-ME, and CGA-LT were 0.4679 (±0.024), 0.1134 (±0.004), and 0.1819 (±0.010), respectively. The larger the StI of a compound was, the higher the comprehensive stability was.

### 2.3. Identification of Degradation Products of CGA, CGA-ME, and CGA-LT

**Figure 6:**
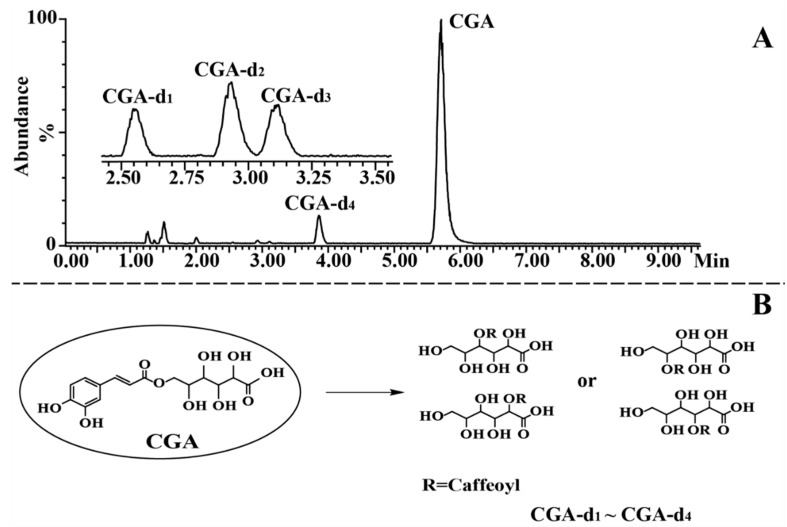
Representative total ion current (TIC) chromatogram of the reacted solution of CGA in the negative ion mode (**A**) and proposed pathways of degradation for CGA (**B**).

**Figure 7:**
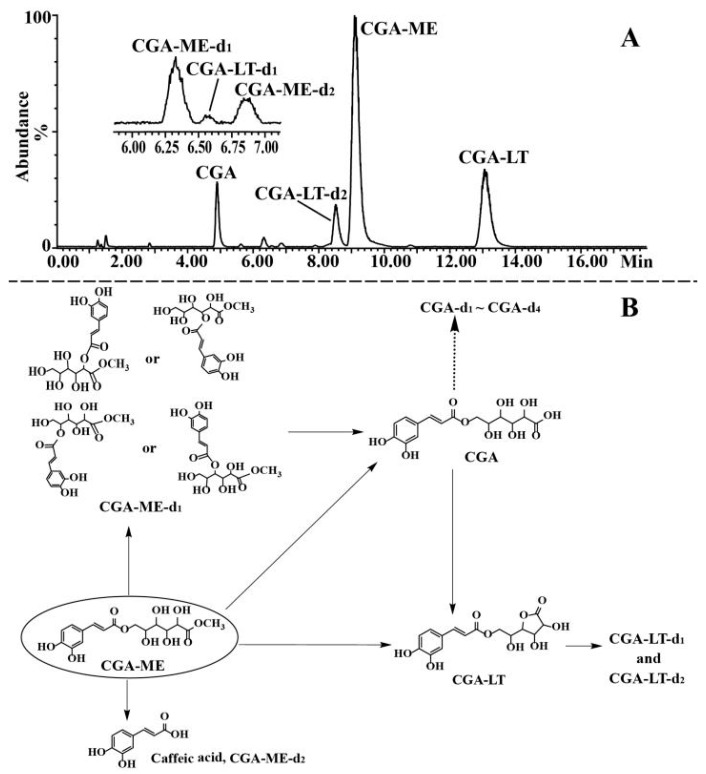
Representative TIC chromatogram of the reacted solution of CGA-ME in the negative ion mode (**A**) and proposed pathways of degradation for CGA-ME (**B**).

**Figure 8:**
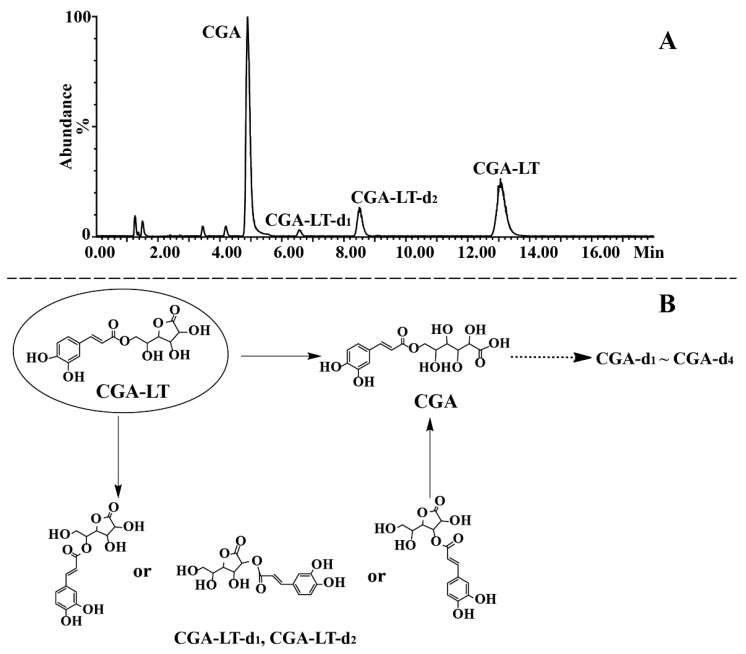
Representative TIC chromatogram of the reacted solution of CGA-LT in the negative ion mode (**A**) and proposed pathways of degradation for CGA-LT (**B**).

Suffering at high temperatures and in alkaline mediums, CGA, CGA-ME, and CGA-LT were subject to similar degradation pathways. Therefore, based on accurate mass information, the degradation products of CGA, CGA-ME, and CGA-LT were characterized by employing the exposed samples of CGA at 80 °C and those of CGA-ME and CGA-LT at pH 8, respectively. Inorganic salt in buffer solution would contaminate MS and deteriorate the MS sensitivity; therefore, ammonia was employed to adjust the pH of the processed sample solution. Given the perfect MS signal of CGA derivatives and degradation products, the negative detection mode was selected. In order to obtain a satisfactory separation of the targeted compounds and their degraded compounds, different column temperatures were employed for the reacted solution of CGA (40 °C), and CGA-ME and CGA-LT (48 °C); therefore, the retention time of CGA in Figure 6 was longer than that in Figure 7 and Figure 8.

CGA showed a quasi-molecular ion at *m*/*z* 357.0839 [M − H]^−^, and its characteristic fragment ions were detected at *m*/*z* 195.0509 [M − H−caffeoyl]^−^, 179.0345 [M − H−gluconic acid residue]^−^, 135.0446 [M − H−gluconic acid residue−CO_2_]^−^, and 129.0189 [M − H−caffeoyl−2H_2_O−CH_2_O]^−^. The detailed MS information of CGA is listed in Table S1. The representative MS and MS^2^ spectra and proposed fragmentation pathways are shown in Figure S2. The main degradation products derived from CGA, CGA-d_1_ to CGA-d_4_, were detected. They had similar MS data and fragment pathways to those of CGA and were identified as isomers of CGA. It can be proposed that CGA underwent a hydrolytic reaction to form caffeic acid and gluconic acid, leading to the production of isomers of CGA by esterification via caffeic acid and one of other four alcohol hydroxyls of gluconic acid [30,32]. The chromatogram of the sample solution and proposed degradation pathways of CGA are shown in Figure 6.

As a methyl-esterified derivative of CGA, CGA-ME indicted a quasi-molecular ion at *m*/*z* 371.0984 [M − H]^−^, whose mass weight was 14 Da larger than that of CGA. The characteristic neutral loss produced ions at *m*/*z* 339.0717 [M − H−CH_3_OH]^−^, 179.0343 [M − H−methyl gluconic acid residue]^−^, 177.0399 [M − H−CH_3_OH−caffeoyl]^−^, and 161.0239 [M − H−methyl gluconic acid]^−^. Then, the consecutive loss of CO_2_ from the ion at *m*/*z* 179.0343 generated an ion at *m*/*z* 135.0446. In Table S1, the detailed MS information of CGA-ME is supplied. Displayed in Figure S3 are the representative MS and MS^2^ spectra and proposed fragmentation pathways. Based on the MS data in Table S1, it was proposed that CGA-ME was degraded into caffeic acid, CGA, and isomers of CGA-ME through a hydrolytic reaction. Further, the lactonized gluconic acid of CGA produced CGA-LT and its isomer. Shown in Figure 7 are the chromatogram of the sample solution and proposed degradation pathways of CGA-ME.

CGA-LT, the lactonized derivative of CGA, exhibited a quasi-molecular ion at *m/z* 339.0715 [M − H]^−^, and its characteristic fragment ions were detected at *m/z* 179.0341, 177.0397, and 161.0236 in the negative ion mode, corresponding to the loss of glucono-*γ*-lactone residue, caffeoyl, and glucono-*γ*-lactone, respectively. Furthermore, the consecutive loss of CO_2_ from the ion at *m/z* 179.0341 generated an ion at *m/z* 135.0443. The detailed MS information of CGA-LT is listed in Table S1. The representative MS and MS^2^ spectra and proposed fragmentation pathways are shown in Figure S4. According to the MS data, it can be deduced that CGA-LT was transformed into isomers of CGA-LT via a hydrolytic reaction or decomposed into CGA through an opening-ring reaction. The chromatogram of the sample solution and proposed degradation pathways of CGA-LT are shown in Figure 8.

## 3. Materials and Methods

### 3.1. Reagents and Materials

CGA, CGA-ME, and CGA-LT were purified by us using D101 macroporous resin, Sephadex LH-20 and ODS column chromatography, and preparative HPLC from the aqueous extract of FE; the compounds were identified by UV, IR, MS, and NMR technologies [14]. Their purities above 95% were determined by UPLC analysis. LC-grade acetonitrile was purchased from Sigma-Aldrich (St. Louis, MO, USA). LC-grade water was produced by a Milli-Q water purification system (Millipore, Massachusetts, MA, USA). Formic acid was bought from Meridian Medical Technologies (MREDA, New York, NY, USA). Phosphoric acid, disodium hydrogen phosphate, potassium dihydrogen phosphate, and dipotassium hydrogen phosphate were bought from DAMAO Chemical Reagent Factory (Tianjin, China).

The phosphate buffer solution at pH 2, 4, 6, and 8 was prepared according to the guidelines released by Chinese pharmacopoeia.

### 3.2. UPLC Analysis

Composed of a column heater, a sample manager, a binary solvent manager, and a photo-diode array (PDA) detector, a Waters Acquity UPLC system (Waters, Milford, MA, USA) was employed to undertake the chromatographic separation on an Acquity UPLC BEH C18 column (3.0 mm × 100 mm, 1.7 μm) with the column heater fixed at 40 °C. The mobile phase was acetonitrile (A) and 0.1% formic acid aqueous solution (B) with an isocratic elution at 8% acetonitrile. The flow rate was 0.3 mL/min. The detection wavelength was 320 nm and the injection volume was 4 μL.

### 3.3. UPLC-Q/TOF MS Analysis

Analysis was performed on a Waters ACQUITY UPLC tandem Xevo G2-S spectrometer (Waters, Milford, MA, USA). The chromatographic condition of UPLC was employed as described in Section 3.2. However, the column temperatures were maintained at 40 °C for CGA analysis and at 48 °C for CGA-ME and CGA-LT. For the MS analysis, nitrogen was used as desolvation gas at a flow rate of 600 L/h for ESI (−). The flow rate of the cone gas was set at 50 L/h. The source and desolvation temperature were fixed at 100 °C and 450 °C, respectively. The capillary voltage was −2.0 kV. The collision energy was 10–60 eV. The spectra were recorded in the range of *m/z* 100–1200 for a full scan. 

### 3.4. Stability Study of Tested Compounds under Different Temperatures and pH Values

CGA, CGA-ME, and CGA-LT (about 1.5 mg) were dissolved in 10 mL of water, respectively. The sample solution (2.5 mL) was incubated at the different temperatures (20, 40, 60, and 80 °C) for 12 h and analyzed following the established UPLC-DAD method to record the concentrations of the tested compounds once per hour. 

CGA, CGA-ME, and CGA-LT (about 3 mg) were each dissolved in 2 mL of water. The sample solution (0.5 mL) was separately mixed with 4.5 mL of buffer solution with a pH of 2, 4, 6, and 8, incubated at 4 °C for 12 h, and analyzed to track the concentration variations of the tested compounds once per hour. In order to guarantee the reliability of results, the stability study of each compound was repeated three times.

CGA, CGA-ME, and CGA-LT (about 0.5 mg) were dissolved in 2 mL of water, respectively. Ammonia solution was added to the solution to adjust the pH at 8. CGA, CGA-ME, and CGA-LT (about 0.5 mg) were each dissolved in 2 mL of water and incubated for 10 h at 80 °C. The processed sample solution was diluted five times with water for UPLC-DAD/ESI-Q-TOF MS analysis. 

### 3.5. Statistical Analysis

The plotting and curve fitting were performed using GraphPad Prism 5.01 software (Graphpad software Inc., La Jolla, CA, USA).

## 4. Conclusions

According to the time–concentration curves as well as SeI and StI of the tested compounds, it was concluded that the integrated stability of CGA was better than that of CGA-ME and CGA-LT. CGA-ME was proved to be the most unstable. Moreover, CGA was inert to the variation of temperature and pH, while CGA-ME and CGA-LT were more susceptible to the variation of temperature. The degradation products and pathways of CGA, CGA-ME, and CGA-LT were identified, as they were characterized by the formation of isomers of the tested compounds via a hydrolytic reaction. Consequently, this study sheds light on the stability of the tested compounds and contributes to improving the manufacturing and quality control of FE and its preparation. 

## Supplementary Materials

The following are available online. Figure S1: Chemical structural formula of CGA, CGA-ME, and CGA-LT; Figure S2: MS (A) and MS^2^ (B) spectra and proposed fragmentation pattern (C) of CGA; Figure S3: MS (A) and MS^2^ (B) spectra and proposed fragmentation pattern (C) of CGA-ME; Figure S4: MS (A) and MS^2^ (B) spectra and proposed fragmentation pattern (C) of CGA-LT; Table S1: Characterization of the degradation products of CGA, CGA-ME, and CGA-LT by UPLC-DAD/ESI-Q-TOF MS.
