# Supplementary material for: Stability Study and Identification of Degradation Products of Caffeoylgluconic Acid Derivatives from Fructus Euodiae
Source: Molecules. 2018 Aug 8;23(8):1975. doi: 10.3390/molecules23081975 (PMC6222684; doi:10.3390/molecules23081975)
Supplement: Supplementary file 1 [file molecules-23-01975-s001.pdf]

Supporting Information

Stability Study and Identification of Degradation Products of Caffeoylgluconic Acid Derivatives from Fructus Euodiae

Huijuan Yu †, Jing Yang *,†, Jiamin Ding, Ying He, Zhenzuo Jiang, Xin Chai * and Yuefei Wang

Tianjin State Key Laboratory of Modern Chinese Medicine, Tianjin University of Traditional Chinese Medicine, Tianjin 300193, China; yuhuijuan_2017@126.com (H.Y.); 13848106684@163.com (J.D.); heying441423@163.com (Y.H.); zhenzuojiang@hotmail.com (Z.J.); wangyf0622@tjutcm.edu.cn (Y.W.)

* Correspondence: yangj0622@tjutcm.edu.cn (J.Y.); chaix0622@tjutcm.edu.cn (X.C.); Tel.: +86-22-27386453 (X.C.)

† These authors contributed equally to this work.

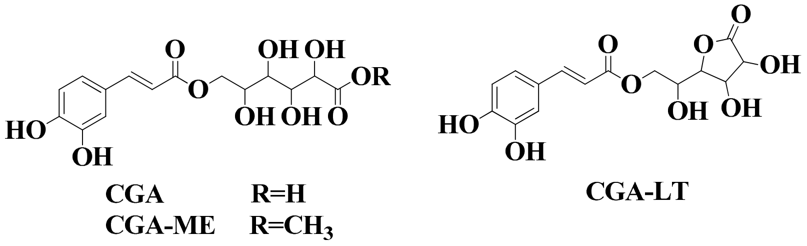

Figure S1. The chemical structural formula of CGA, CGA-ME and CGA-LT.

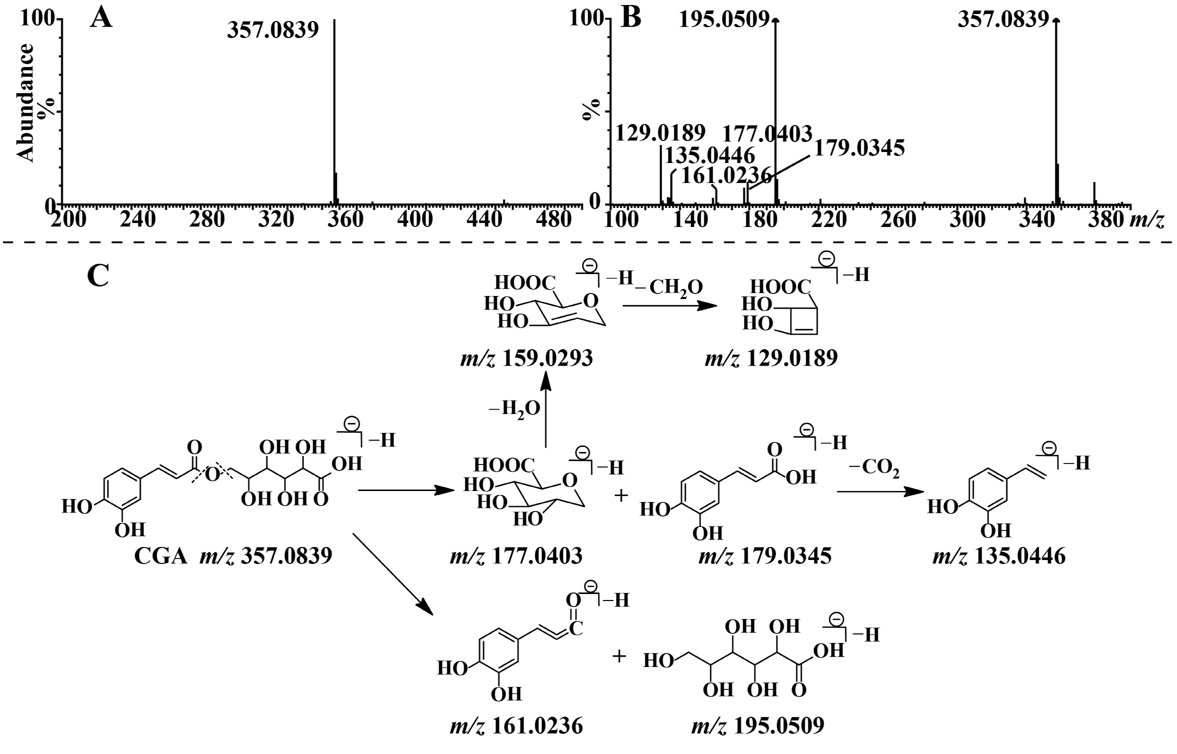

Figure S2. MS (A) and MS[2] (B) spectra, and the proposed fragmentation pattern (C) of CGA.

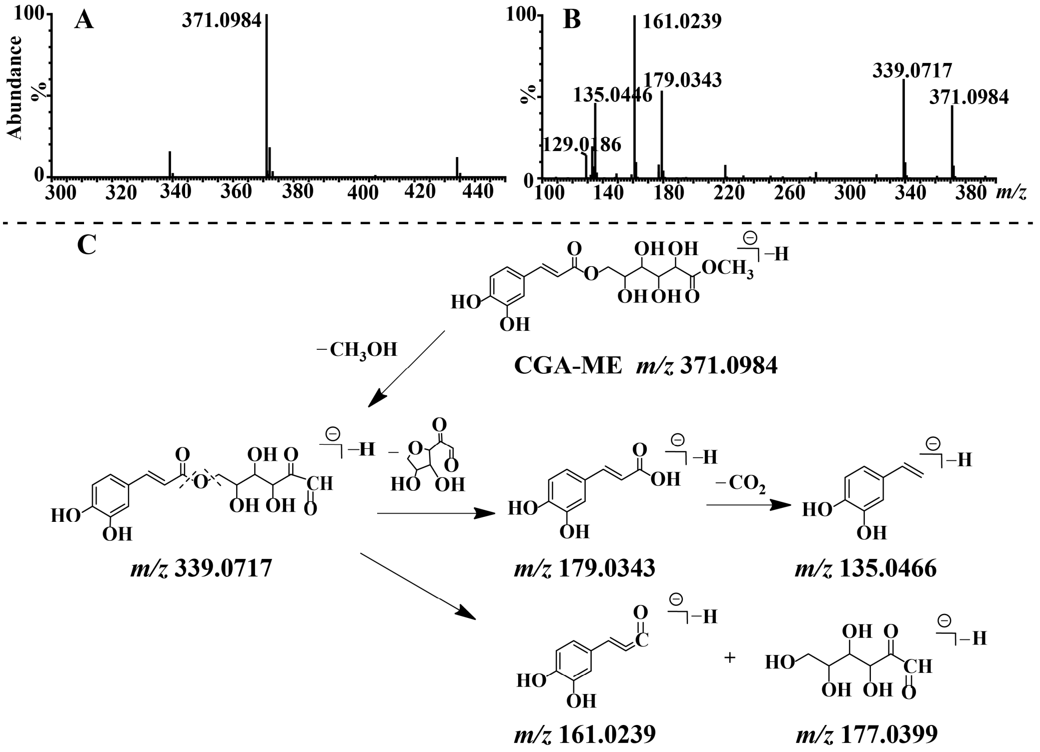

Figure S3. MS (A) and MS² (B) spectra, and the proposed fragmentation pattern (C) of CGA-ME.

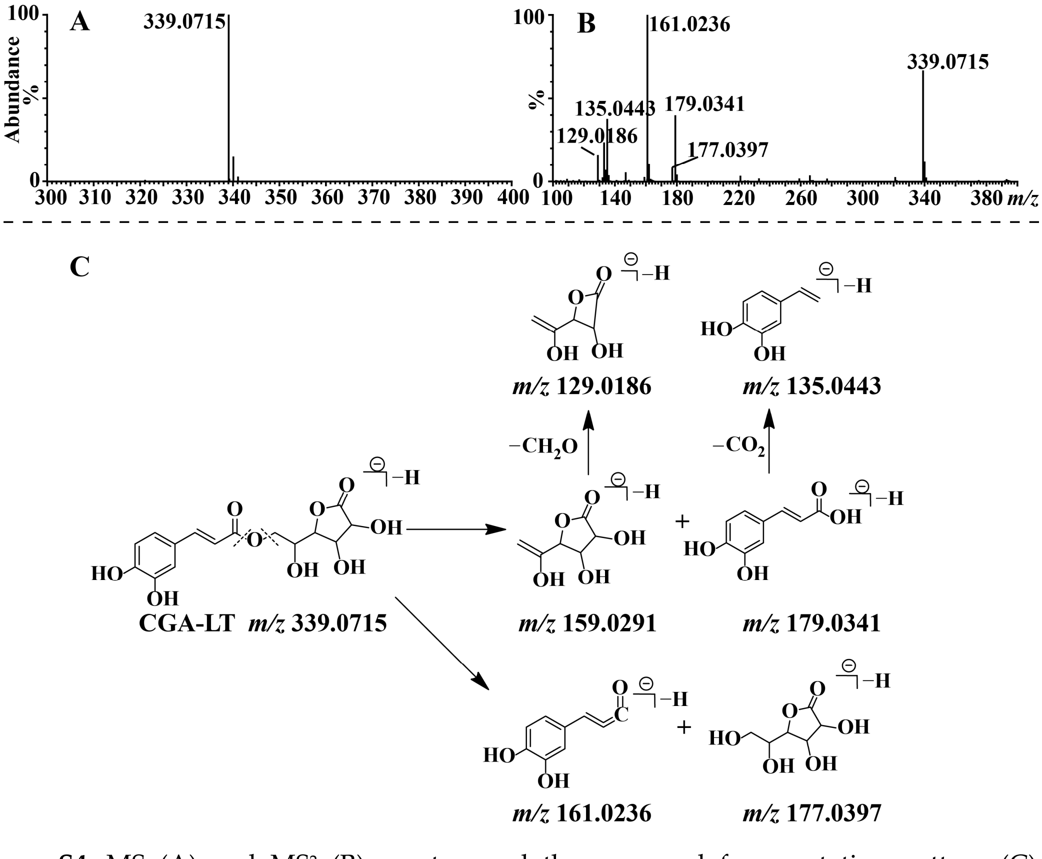

Figure S4. MS (A) and MS² (B) spectra, and the proposed fragmentation pattern (C) of CGA-LT.

Table S1. Characterization of the degradation products of CGA, CGA-ME and CGA-LT by UPLC-DAD/ESI-Q-TOF MS.

Compounds	t_R (min)	$[M–H]^-$	MS^2	Identification
CGA–d_1	2.55	357.0820	195.0503 $[M–H–caffeoyl]^-$	Isomer of CGA
			179.0342 $[M–H–gluconic\ acid\ residue]^-$	
			135.0443 $[M–H–gluconic\ acid\ residue–CO_2]^-$	
			129.0187 $[M–H–caffeoyl–2H_2O–CH_2O]^-$	
CGA–d_2	2.93	357.0822	195.0504 $[M–H–caffeoyl]^-$	Isomer of CGA
			179.0345 $[M–H–gluconic\ acid\ residue]^-$	
			135.0445 $[M–H–gluconic\ acid\ residue–CO_2]^-$	
			129.0188 $[M–H–caffeoyl–2H_2O–CH_2O]^-$	
CGA–d_3	3.12	357.0822	195.0504 $[M–H–caffeoyl]^-$	Isomer of CGA
			179.0343 $[M–H–gluconic\ acid\ residue]^-$	
			135.0444 $[M–H–gluconic\ acid\ residue–CO_2]^-$	
			129.0189 $[M–H–caffeoyl–2H_2O–CH_2O]^-$	
CGA–d_4	3.87	357.0825	195.0506 $[M–H–caffeoyl]^-$	Isomer of CGA
			179.0343 $[M–H–gluconic\ acid\ residue]^-$	
			135.0445 $[M–H–gluconic\ acid\ residue–CO_2]^-$	
			129.0188 $[M–H–caffeoyl–2H_2O–CH_2O]^-$	
CGA	5.70	357.0839	195.0509 $[M–H–caffeoyl]^-$	CGA
			179.0345 $[M–H–gluconic\ acid\ residue]^-$	
			135.0446 $[M–H–gluconic\ acid\ residue–CO_2]^-$	
			129.0189 $[M–H–caffeoyl–2H_2O–CH_2O]^-$	
CGA-ME → CGA	4.90	357.0819	195.0509 $[M–H–caffeoyl]^-$	CGA
			179.0341 $[M–H–gluconic\ acid\ residue]^-$	
			135.0443 $[M–H–gluconic\ acid\ residue–CO_2]^-$	
			129.0186 $[M–H–caffeoyl–2H_2O–CH_2O]^-$	

CGA-ME–d_1	6.33	371.0988	339.0713 [M–H–CH_3OH]$^-$<br>179.0343 [M–H–methyl gluconic acid residue]$^-$<br>177.0398 [M–H–CH_3OH–caffeoyl]$^-$<br>161.0237 [M–H–methyl gluconic acid]$^-$<br>135.0445 [M–H–methyl gluconic acid residue–CO_2]$^-$	Isomer of CGA-ME
CGA-LT–d_1	6.58	339.0714	–	Isomer of CGA-LT
CGA-ME–d_2	6.89	179.0342	–	Caffeic acid
CGA-LT–d_2	8.55	339.0712	179.0341 [M–H–glucono-γ-lactone residue]$^-$<br>177.0397 [M–H–Caffeoyl]$^-$<br>161.0236 [M–H–glucono-γ-lactone]$^-$<br>135.0443 [M–H–glucono-γ-lactone residue–CO_2]$^-$<br>129.0185 [M–H–caffeic acid–CH_2O]$^-$	Isomer of CGA-LT
CGA-ME	9.09	371.0984	339.0717 [M–H–CH_3OH]$^-$<br>179.0343 [M–H–methyl gluconic acid residue]$^-$<br>177.0399 [M–H–CH_3OH–caffeoyl]$^-$<br>161.0239 [M–H–methyl gluconic acid]$^-$<br>135.0446 [M–H–methyl gluconic acid residue–CO_2]$^-$	CGA-ME
CGA-ME → CGA-LT	13.07	339.0715	179.0342 [M–H–glucono-γ-lactone residue]$^-$<br>177.0399 [M–H–caffeoyl]$^-$<br>161.0237 [M–H–glucono-γ-lactone]$^-$<br>135.0443 [M–H–glucono-γ-lactone residue–CO_2]$^-$<br>129.0185 [M–H–caffeic acid–CH_2O]$^-$	CGA-LT

CGA-LT → CGA	4.90	357.0838	195.0505 [M–H–caffeoyl]$^-$<br>179.0341 [M–H–gluconic acid residue]$^-$<br>135.0443 [M–H–gluconic acid residue–CO_2]$^-$<br>129.0186 [M–H–caffeoyl–$2H_2O$–CH_2O]$^-$	CGA
CGA-LT–d_1	6.56	339.0728	179.0345 [M–H–glucono-γ-lactone residue]$^-$<br>177.0399 [M–H–caffeoyl]$^-$<br>161.0238 [M–H–glucono-γ-lactone]$^-$<br>135.0445 [M–H–glucono-γ-lactone residue–CO_2]$^-$<br>129.0186 [M–H–caffeic acid–CH_2O]$^-$	Isomer of CGA-LT
CGA-LT–d_2	8.50	339.0710	179.0340 [M–H–glucono-γ-lactone residue]$^-$<br>177.0395 [M–H–caffeoyl]$^-$<br>161.0234 [M–H–glucono-γ-lactone]$^-$<br>135.0442 [M–H–glucono-γ-lactone residue–CO_2]$^-$<br>129.0184 [M–H–caffeic acid–CH_2O]$^-$	Isomer of CGA-LT
CGA-LT	13.01	339.0715	179.0341 [M–H–glucono-γ-lactone residue]$^-$<br>177.0397 [M–H–caffeoyl]$^-$<br>161.0236 [M–H–glucono-γ-lactone]$^-$<br>135.0443 [M–H–glucono-γ-lactone residue–CO_2]$^-$<br>129.0186 [M–H–caffeic acid–CH_2O]$^-$	CGA-LT
